# Preparation and *In Vitro/Ex Vivo* Evaluation of Moxifloxacin-Loaded PLGA Nanosuspensions for Ophthalmic Application

**DOI:** 10.3797/scipharm.1204-16

**Published:** 2013-02-04

**Authors:** Meetali Mudgil, Pravin K. Pawar

**Affiliations:** Chitkara College of Pharmacy, Chitkara University, Chandigarh-Patiala Highway, Rajpura, Patiala, Punjab, 140401, India.

**Keywords:** Ocular, Goat cornea, Nanosuspension, Korsemeyer-Peppas, Moxifloxacin, PLGA

## Abstract

The aim of the present investigation was to prepare a colloidal ophthalmic formulation to improve the residence time of moxifloxacin. Moxifloxacin-loaded poly(dl-lactide-*co*-glycolide) (PLGA) nanosuspensions were prepared by using the solvent evaporation technique. The nanosuspensions were characterised physically by using different techniques like particle size, zeta potential, FTIR, DSC, and XRD analysis. *In vitro* and *ex vivo* studies of nanosuspensions were carried out using a modified USP dissolution apparatus and all-glass Franz diffusion cells, respectively. The antibacterial activities of the nanosuspension and marketed formulations were performed against *S. aureus* and *P. aeroginosa*. The moxifloxacin-loaded PLGA nanosuspensions showed uniform particle size, ranging between 164–490 nm with negative zeta potential for all batches. The percentage entrapment efficiency of the drug-loaded nano-suspension was found to be between 84.09 to 92.05%. *In vitro* drug release studies suggest that all of the formulations showed extended drug release profiles and follow Korsemeyer-Peppas release kinetics. *In vitro* corneal permeability was found to be comparable with that of the marketed formulation across isolated goat cornea, indicating the suitability of the nanosuspension formulation in the ophthalmic delivery of moxifloxacin. The optimised nano-suspension was found to be more active against *S. aureus* and *P. aeruginosa* compared to the marketed eye drops.

## Introduction

The eyes are among the most readily accessible organs in terms of drug delivery. The physiological constraints of the eye render this organ impervious to foreign substances, thus presenting a constant challenge to the pharmaceutical scientist to circumvent the protective barriers of the eye without causing permanent tissue damage [1]. Basically, ocular infections are treated by using topical application of antibiotics in the form of eye drops. About 90 % of the dose applied topically from such solutions is lost due to precorneal losses (lacrimation and drainage) which leads to poor aqueous availability, so frequent dosings are required for the instillation to achieve an adequate level and therapeutic effect [2]. To overcome these problems, various novel drug delivery systems for ophthalmic applications such as ocular inserts, collagen shields, colloidal, or particulate systems like nanoparticles, nanocapsules, niosomes, and liposomes have been developed to prolong the residence time and to improve the bioavailability. Nanoparticulate systems have received considerable attention over the past 25 years due to their advantages compared to other drug delivery systems. These advantages include: targeted delivery of drugs to the specific site to minimize toxicity; improved bioavailability by reducing fluctuations in drug levels; improved stability of drugs against enzymatic degradation; sustained and controlled release effect that reduces dosing frequency with improved patience compliance; and the ease of administration through various routes including oral, nasal, pulmonary, intraocular, parenteral, and transdermal. Considering the above advantages, nanotechnology has been used in ocular drug delivery to achieve extended drug release in the management of external inflammatory/autoimmune ocular diseases [3, 4].

Moxifloxacin is a fourth-generation fluoroquinolone with a methoxy group in the C-8 position and a bulky C-7 side chain [5]. This fourth-generation fluoroquinolone has *in vitro* activity similar to that of ciprofloxacin and ofloxacin against Gram-negative bacteria like *Pseudomonas aeruginosa,* but enhanced activity against Gram-positive bacteria including *S. aureus*. The bactericidal activity of moxifloxacin is mediated by the inhibition of DNA gyrase (topoisomerase II) and topoisomerase IV, essential enzymes involved in bacterial DNA replication, transcription, repair, and recombination [6]. Moxifloxacin is more effective than ciprofloxacin or levofloxacin in experimental keratitis in rabbits [7]. Penetration of moxifloxacin into the inflamed ocular tissue of rabbits has been found to be better than ciprofloxacin, lomefloxacin, ofloxacin, or levofloxacin [8].

On the basis of a literature survey, poly(lactide-*co*-glycolide) (PLGA) [9–12], poly(lactide-*co*-glycolide-leucine) (PLDL) [13], Eudragit RL-100 [14, 15], Eudragit RS-100 [16–19], Eudragit E-100 [20], Eudragit S-100 [21], and Chitosan [22] are also used in ophthalmic drug formulations. Among them, PLGA is the most suitable candidate for sustained drug delivery as it is a biodegradable and biocompatible polymer that degrades hydrolytically into an oligomer and monomer, lactic acid, and glycolic acid, which are non-toxic in nature [23]. Also, PLGA is approved for use in drug delivery applications by the Food and Drug Administration (FDA) [24]. For this reason, PLGA is used in a nanoparticulate formulation for ophthalmic drug delivery.

Taking the above information in view, the purpose of the current study was to formulate and evaluate a new colloidal system of moxifloxacin-loaded PLGA nanoparticles for ophthalmic delivery, which would be capable of prolonging the contact time, thereby potentially enhancing the intra-corneal delivery of ophthalmic API.

## Experimental

### Materials

Moxifloxacin hydrochloride was received as a gift from Ind-Swift Laboratories Ltd. (Derabassi, India). Poly(dl-lactide-*co*-glycolide) (PLGA) (75:25) was obtained from Sigma Aldrich Chemie GmbH (Steinheim, Germany). Tween 80 was procured from Loba Chemi Pvt Ltd. (Mumbai, India). All other chemicals used were of analytical grade.

### Preparation of nanoparticles

Moxifloxacin-loaded PLGA nanoparticles were prepared by a nanoprecipitation technique with a slight modification to the previously reported process [11]. To prepare the nanoparticle suspensions, 0.1 gm of drug and 0.1 gm to 0.5 gm of PLGA were dissolved in 10 ml acetone to form a homogenous mixture. Then the prepared solution of the drug and polymers was dropped by using a syringe (26 gauge) with a constant speed (0.5ml/min) into distilled water (20 ml) containing Tween 80 (0.2% w/v). The mixture was homogenized using a magnetic stirring at a constant agitation speed of 1400 rpm for 2 hrs. An excess amount of acetone was evaporated by air-drying and the final volume of the suspension was collected. The resulting suspension was further sonicated using an ultrasonic probe sonicator (PCI analytics, Mumbai, India).

### Characterization of the Nanosuspension

#### Determination of drug entrapment efficiency

The moxifloxacin nanosuspension (10 ml) was centrifuged at 19000 rpm, 10°C using the Cooling Centrifuge Instrument (24 BL model, Remi, Mumbai, India) for 2 h. The supernatant was separated out; the absorbance was measured for the free drug content using a UV/Visible spectrophotometer at 288 nm (Systronics, 2701, Mumbai, India). The entrapment efficiency of the moxifloxacin nanosuspension was determined by subtracting the free drug amount from the initial added amount of the drug [25]. The entrapment efficiency (EE %) could be calculated by the following equation

$$\text{Entrapment}\;\text{efficiency}\;\left(\text{EE}\%\right)=\frac{\text{Initial}\;\text{drug}-\text{Free}\;\text{drug}}{\text{Initial}\;\text{drug}}\times 100$$

#### Particle size and Zeta potential measurement

The mean particle size for the formulations was determined by photon correlation spectroscopy (PCS) with a Zetasizer Nano ZS-90 (Malvern Instruments Ltd, Worcestershire, UK). The reading was carried out at a 90° angle to the incident beam at 25°C using a proper dilution with filtered water (0.5 micrometer filter). The conductivity of all samples was fixed to 2.43 ms/cm. The zeta potential was determined by a laser-doppler-anemometer coupled with a Zetasizer Nano ZS-90 (Malvern Instruments Ltd.; UK). All experiments were done in triplicate.

#### Transmission electron microscopy (TEM)

Transmission electron microscopy (TEM) was performed for the morphological evaluation of the nanoparticles. The nanoparticle suspension (5–10 μL) was dropped onto carbon-coated copper grids for viewing by transmission electron microscopy (Hitachi H-7500, Tokyo, Japan). Imaging viewer software was used to perform the image capture and analysis.

#### Fourier Transform Infrared spectroscopy (FTIR)

The FTIR spectra of moxifloxacin, PLGA, and the physical mixture in the 1:1 drug:polymer ratio and freeze-dried nanosuspension were carried out in the range of 4000–400 cm^−1^ by FTIR-IR spectroscopy (Perkin Elmer Model 1600 Spectrum BX FT-IR Spectrophotometer, Wellesley, MA, US) using the potassium bromide (KBr) disk technique.

#### X-ray powder diffraction (PXRD)

The crystalline state of the drug in the polymer sample was evaluated by X-ray powder diffraction (PXRD) analysis. The X-ray powder diffraction patterns of moxifloxacin, PLGA, and the physical mixture in the 1:1 drug:polymer ratio and freeze-dried nanosuspension (MN4) were recorded with the XPERT-PRO X-ray diffractometer using the PRS measurement program using Ni-filtered, CuKα radiation with a voltage of 45 kV, and a current of 40 mA. The instrument was operated in the continuous scanning speed of over a 2θ range of 5° to 49°.

#### Differential scanning calorimetry (DSC)

DSC studies of moxifloxacin, PLGA, and the physical mixture in the 1:1 drug:polymer ratio and freeze-dried nanosuspension were carried out. Samples were separately sealed in aluminium cells and set in the Perkin-Elmer DSC 7 apparatus (Perkin-Elmer DSC 7, Waltham, MA, US) between 50°C and 350°C. Thermal analysis was performed at a heating rate maintained at 10°C per minute in a nitrogen atmosphere. An empty pan of alumina was used as the reference in each case.

#### In-vitro drug release

The *in vitro* drug release study of the nanosuspension was performed in the modified USP dissolution apparatus 1 containing a two-sided open glass cylinder. A pre-soaked dialysis membrane (cut off 12000–14000) A (Himedia, Mumbai) was adapted to the terminal portion of the glass cylinder. The moxifloxacin nanosuspension (2 ml) was accurately placed into the glass cylinder from the open side and this cylinder was fixed on the stirrer. The stirrer was suspended in a 100 ml dissolution of simulated tear fluid (pH 7.4) medium maintained at 37ºc ± 5ºc at 100 rpm, so that the dialysis membrane-fixed cylinder end just touched the receptor medium surface. The composition of simulated tear fluid (pH 7.4) was as follows: Nacl-0.672 gm, NaHCO_3_-0.200 gm, CaCl_2_-0.008 gm and the volume was made up to 100 ml with distilled water. The samples were withdrawn at specified time intervals with volume replacement. The withdrawn samples were analysed after proper dilution using simulated tear fluid at pH 7.4 for drug content, by measuring absorbance at 288 nm in the UV/Visible spectrophotometer. All the experiments were conducted in triplicate.

#### Ex vivo transcorneal permeation study

*Ex vivo* transcorneal permeation studies were carried out by putting the moxifloxacin nanosuspension/ marketed eye drop (1 ml) on a freshly excised goat cornea. The fresh, whole eyeballs of goats were obtained from a local butcher’s shop and transported to the laboratory chilled in normal saline (4°C). The cornea was then carefully excised along with 2 to 4 mm of surrounding scleral tissue and was washed with normal saline until the washing was free from protein. The excised cornea was fixed between the clamped donor and receptor compartments of an all-glass modified Franz diffusion cell in such a way that its epithelial surface faced the donor compartment. The corneal area available for diffusion was 0.50 cm^2^. The receptor compartment was filled with 10 ml freshly prepared simulated tear fluid (pH 7.4), and all air bubbles were expelled from the compartment. The aliquot (1 ml) of the prepared nanosuspension/ marketed eye drop was placed on the cornea and the opening of the donor cell was sealed with a glass cover slip; the receptor fluid was kept at 37°C with constant stirring using a Teflon-coated magnetic stir bead. The permeation study was carried out for 4 h, and samples were withdrawn from the receptor and analysed for moxifloxacin content by measuring absorbance at 288 nm in a UV/Visible spectrophotometer (Systronics, Mumbai, India). At the end of the experiment, each cornea (freed from adhering sclera) was weighed, soaked in 1 ml methanol, dried overnight at 80°C, and reweighed. From the difference in weights, corneal hydration was calculated [27, 28].

#### Microbiological studies

The microbiological studies ascertained the biological activity of the optimized formulation (MN4) and of the marketed eye drops against microorganisms (*Pseudomonas aeruginosa* and *Staphylococcus aureus*) [29]. A layer of nutrient agar (20 ml) seeded with the test microorganism (0.2 ml) was allowed to solidify in the Petri plate. Cups were made on the solidified agar layer with the help of a sterile borer at 4 mm diameter. Then, a volume of the formulations (MN4 and the marketed eye drop) containing an equivalent amount of the drug was separately poured into the cups. After keeping the Petri plates at room temperature for 4 h, the plates were incubated at 37°C for 24 h. The zones of inhibition were obtained. The diameter of the zone of inhibition was measured by an antibiotic zone finder. The observation and measurements were taken in triplicate.

#### Stability study of nanosuspension

An optimised nanosuspension batch was chosen to perform the stability study of the nanosuspension. Samples were stored in glass vials for six months at room temperature (20°C) and at 4°C in a freezer. After six months, samples were visually observed for any sedimentation, and entrapment efficiency was calculated. The particle size and zeta potential of the optimised formulation was also measured after six months. The number of observations was taken in triplicate.

## Results and discussion

### Drug entrapment efficiency (EE %)

The entrapment efficiency of all of the nanosuspension formulations was found to be above 84% (Table 1). It is evident from Table 1, that the percentage entrapment efficiency was affected by the drug:polymer ratio. The results revealed that the entrapment efficiency of all formulations was increased with increasing concentration of the polymer in the formulations, except MN5, which might be due to the saturation effect of the polymer concentration. The result was in accordance with previously published work [3].

### Particle size and Zeta potential measurements

The effect of PLGA concentration on the particle size of different formulations is depicted in Table 1. The formulations MN1, MN2, MN3, and MN5 provide higher particle size values. On the basis of small particle size i.e. 164.80 nm and a lower polydispersity index (0.158 nm), the formulation MN4 was the optimised formulation in comparison with the remaining formulations. Larger particle size will induce rapid tear production which leads to rapid drainage of the instilled dose and therefore reduced bioavailability. The results of particle size were found to be in accordance with previously published work [30, 31].

Zeta potential is an important parameter to analyse the long-term stability of nanoparticles. Generally higher zeta potential values, both (+) or (−), indicate long-term stability because of electrostatic repulsion between particles with the same charges, which avoids aggregation [32–34]. The zeta potential of the optimised nanoparticles was found to be around −54.03 mV which indicated a stable formulation. The negative charge of PLGA was due to ionization of the carboxylic end group.

### Transmission electron microscopy (TEM)

**Fig. 1:**
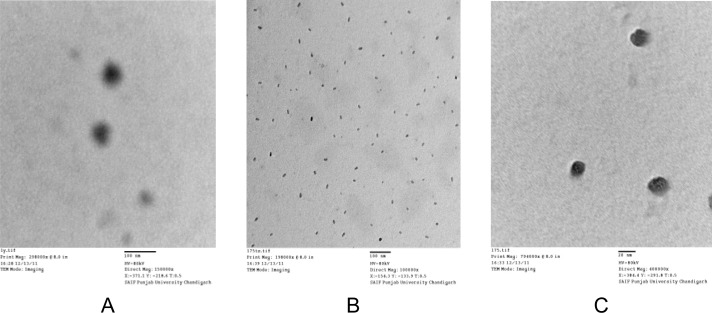
TEM micrograph of optimised nanosuspension (MN4); A: at 150000×, B: at 100000×, C: at 400000×.

The morphology of nanoparticles was determined by Transmission electron microscopy (TEM). TEM gives information about the structure and size of nanoparticles. Prepared nanoparticles were found to be spherical in shape as shown in Fig. 1. A narrow range of particle size was observed for nanoparticles, which exhibited a monodisperse distribution.

### Fourier Transform Infrared spectroscopy (FTIR)

**Fig. 2:**
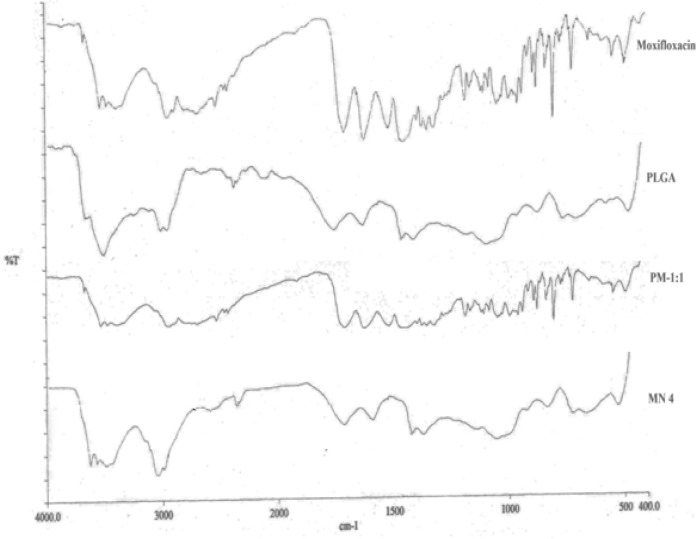
FTIR Spectra of Moxifloxacin, PLGA, Physical mixture of Moxifloxacin and PLGA (PM-1:1), Freeze-dried nanosuspension batch (MN4).

The FTIR spectra of moxifloxacin, PLGA, and the physical mixture in the 1:1 drug:polymer ratio and freeze-dried nanosuspension (MN4) were found. The FTIR spectra of moxifloxacin showed characteristic peaks at 1706 cm^−1^ due to carboxylic acid C=O stretching, C−N stretching at 1320 cm^−1^, aromatic C=C stretching at 1622 cm^−1^, 1518 cm,^−1^ and 1451 cm^−1^, and C−H bending for the substituted benzene at 1875 cm^−1^ along with the characteristic peak of PLGA at 1749 cm^−1^ (Fig. 2). The FTIR study concluded that all of the drug, polymer, physical mixture, and nanosuspension exhibited the characteristic bands which confirm that there is no interaction between PLGA and moxifloxacin.

### Powder X-ray diffractometry (PXRD)

**Fig. 3:**
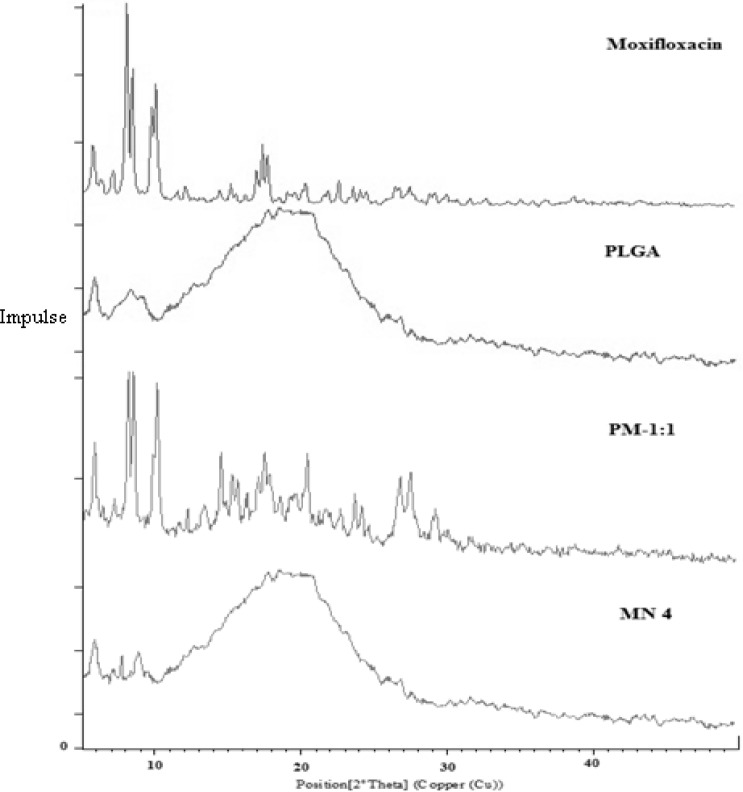
XRD of Moxifloxacin, PLGA, Physical mixture of Moxifloxacin and PLGA (PM-1:1), Freeze-dried nanosuspension batch (MN4).

The X-ray diffractogram of pure moxifloxacin (Fig. 3) showed sharp crystalline peaks, concluding the crystallinity of moxifloxacin. However, the absence of crystalline peaks in PLGA confirms the amorphous nature of the polymer. The X-ray diffractogram of the pure drug and PLGA was compared with that of the physical mixture and the freeze-dried nanosuspension formulation. The X-ray diffractogram of moxifloaxcin showed distinct peaks at 8.0, 8.4, 10.0, and 17.3 at 2θ. The absence of distinct diffraction peaks in the nanosuspension prepared with PLGA suggests amorphization or solid solvation in the amorphous carrier [16, 19].

### Differential scanning calorimetry (DSC)

**Fig. 4:**
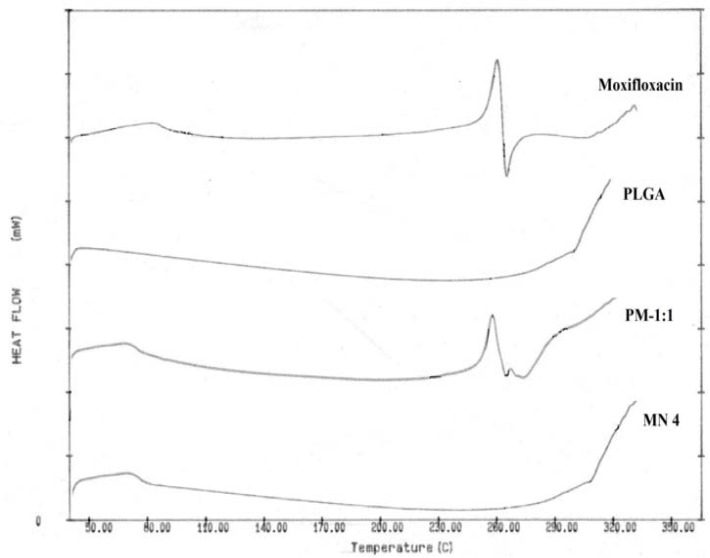
DSC thermograms of Moxifloxacin, PLGA, Physical mixture of moxifloxacin and PLGA (PM-1:1), Freeze-dried nanosuspension batch (MN4).

Degradation of the endotherm of moxifloxacin was observed at 260°C, in DSC of the pure drug and physical mixture of the drug with PLGA. (Fig. 4) However, no distinct peak was observed in the MN4 formulation because of entrapment of the drug in the polymer matrix and owing to the decreased crystallinity in the formulation or drug solvation in the amorphous carrier. These findings are in accordance with the previously reported literature [13].

### In-vitro drug release

**Fig. 5:**
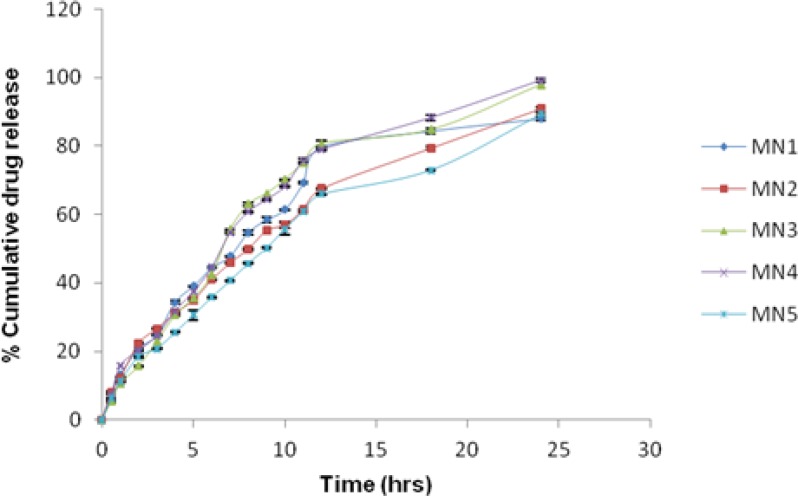
Comparative *in vitro* release profile of moxifloxacin nanosuspension batches. (Mean± SD., n=3)

The formulation MN4 showed maximum cumulative release (%) i.e. 99.17 ± 0.42 at the end of 24 h as compared with other nanosuspension formulations. The release profile (Fig. 5) showed a biphasic release pattern: initial fast release followed by a slow release phase (extended release). The drug release pattern of all the formulations had a biphasic mechanism i.e. the first 10 hours the formulation showed immediate release (burst effect) due to the fraction of drug which was weakly absorbed or bound to the surface of nanoparticles and then followed by extended release which is the effect of the PLGA-entrapped fraction of the drug. The effect of PLGA also contributed to the extended release of the drug from the nanoparticles suspension. An initial fast release pattern is beneficial in terms of antibacterial activity as it helps to achieve the therapeutic concentration of the drug in minimal time, followed by slow release to maintain and sustain the controlled release effect [3, 13]. The *in vitro* release study suggested that the MN4 formulation provided a higher drug release profile as compared to the other formulation of nanosuspension. This might be due to a smaller particle size with a lower polydispersity index, which could help to enhance the dissolution and permeation profile.

### In vitro release kinetics

The drug release data obtained from various *in vitro* release experiments were subjected to various kinetics equations to evaluate the drug release mechanism and kinetics [35]. The kinetics models used were zero order (as the cumulative amount of drug release versus time), first order (as the log cumulative percentage of drug remaining versus time), Higuchi model (as the cumulative percentage of drug released versus square root of time), and Korsemeyer-Peppas (as the log cumulative percentage of drug release versus log time). The *in vitro* release kinetics of all the moxifloxacin-loaded PLGA formulations were studied. The regression coefficients (r^2^) for all the nanosuspension batches using different kinetics equations are listed in Table 2. The kinetics data showed that *in vitro* release from moxifloxacin-loaded PLGA nanoparticles is best explained by the Korsemeyer-Peppas (K-P) model (R=0.979, with value of n∼0.686). The values of n for all nanosuspension batches as per K-P model were found to be between 0.45 and 0.89, which indicate that drug release from the nanosuspensions follow analomous behaviour, where swelling, diffusion, and erosion may play an important role.

### Ex vivo transcorneal permeation study

*Ex vivo* transcorneal permeation studies compared the corneal permeation characteristics of the nanosuspension formulation. The optimised formulation showed higher permeation across the freshly excised goat cornea (47.86% ± 0.14) in 4 h as compared with the marketed eye drop (Moxicip) as shown in Table 3. This increase in permeation through nanoparticles across the cornea is due to the agglomeration of nanoparticles as a depot near the cornea from which the drug is slowly delivered to the precorneal area [3, 29]. Corneal hydration was also calculated to evaluate the damage to corneal tissue. Corneal hydration of all of the formulations was between 78 to 80%, which indicated that the formulations did not cause any damage to the corneal tissue. Normal cornea has a hydration level (HL) of 75 to 80% [36]. An alteration in this level shows damage to the endothelium or epithelium.

### Microbiological studies

**Fig. 6:**
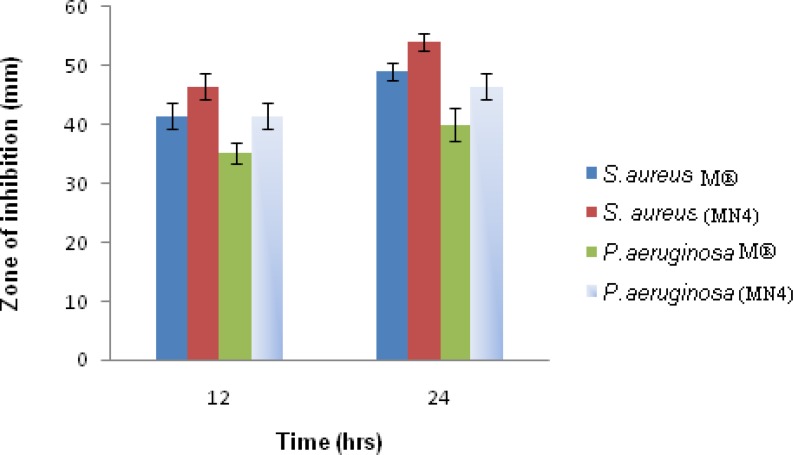
Diameter of zones of inhibition (± standard deviation) for marketed eye drops (M^®^) and the optimised nanosuspension formulation (MN4) using *S. aureus* and *P. aeruginosa.*

In the antimicrobial study, the agar plate method was used and the plate contained two wells in each plate. Secondly, the plates were kept at 4 °C for microbial diffusion purposes, and 37°C incubation was carried out for optimum microorganism growth. Microbiological studies were carried out to compare the antibacterial efficacy of the MN4 nanosuspension with that of Moxicip (M^®^). The diameters of the zones of inhibition are shown in Fig. 6. The zone of inhibition in M^®^ against *Staphylococcus aureus* was obtained 41.5 ± 2.12 and 49.0 ± 1.41 mm at 12 h and 24 h, respectively. However, the zone of inhibition of MN4 was observed 46.5 ± 2.12 and 51.0 ± 1.41 mm at 12 h and 24 h, respectively. The diameter of the zone of inhibition by M^®^ was 35.25 ± 1.70 mm and 40.0 ± 2.83 mm at 12 h and 24 h compared with 41.5 ± 2.12 and 46.5 ± 2.12 mm for MN4 at 12 h and 24 h against *Pseudomonas aeruginosa.* The study suggests that MN4 showed higher antibacterial activity against *Staphylococcus aureus* as compared with M^®^ eye drops. It was concluded that the formulation is more active against *Staphylococcus aureus* compared to *Pseudomonas aeruginosa* and has prolonged microbial efficacy of the nanosuspension (MN4) compared with the marketed eye drops (Moxicip) [37, 38].

### Stability study of nanosuspension

The optimised nanosuspension batch (MN4) was stored in glass vials for six months at room temperature (20°C) and at 4°C in a freezer, and the results showed that the particle size changed from 168–174 nm, and the zeta potential was −54mV to −51mV after six months. No sedimentation occurred in the moxifloxacin nanosuspension and there was a negligible change in entrapment efficiency of the nanoparticles after six months. It can be inferred that the prepared nanosuspension was stable after six months of storage at room temperature and at 4°C in a freezer.

## Conclusions

The study concluded that moxifloxacin-loaded PLGA nanoparticle suspensions were prepared successfully using the solvent evaporation technique. The optimum particle size and higher zeta potential values indicate that the formulations were quite stable with satisfactory drug release. The *in vitro* drug release from all formulationswere biphasic in nature, i.e. first immediate release followed by extended release. The DSC, FTIR, and XRD studies were supportive by providing evidence of the absence of drug-polymer interactions. The optimised formulation of the moxifloxacin nanosuspension showed maximum *in vitro* transcorneal permeation through freshly excised goat cornea and prolonged microbial efficacy against *S. aureus* and *P. Aeruginosa,* compared with the marketed eye drop. Finally, it was concluded that the PLGA-loaded moxifloxacin nanosuspension can be used as a promising ocular drug delivery carrier. With respect to more comments, the *in vivo* efficacy of the abovesaid formulation requires further pre-clinical study.

## Figures and Tables

**Tab. 1.** Physical characterization of formulated moxifloxacin nanosuspension batches. (Mean± SD., n=3)

**Formulation code**	**Drug:Polymer ratio (w/w)**	**Particle size (nm ± SD)**	**Poly-dispersity index**	**Entrapment efficiency (% ± SD)**	**Zeta potential (mV ± SD)**
MN1	1:1	486.10 ± 10.73	0.472 ± 0.03	84.09±0.57	−48.93 ± 0.35
MN2	1:2	324.07 ± 18.44	0.334 ± 0.08	87.58±0.55	−50.57 ± 0.57
MN3	1:3	218.93 ± 10.05	0.259 ± 0.06	89.58±0.51	−51.10 ± 0.46
MN4	1:4	164.80 ± 08.19	0.158 ± 0.03	92.65±0.52	−54.03 ± 0.25
MN5	1:5	490.10 ± 10.55	0.556 ± 0.04	86.78±0.50	−47.37 ± 0.60

**Tab. 2.** Release kinetics of *in vitro* drug release from Moxifloxacin-loaded PLGA nanoparticles.

**Formulation Code**	**r^2^**			
	**Zero-order**	**First-order**	**Higuchi**	**Korsemeyer-Peppas**
MN1	0.867	0.961	0.967	0.978
MN 2	0.950	0.988	0.990	0.992
MN 3	0.956	0.948	0.955	0.964
MN 4	0.888	0.935	0.970	0.979
MN 5	0.938	0.977	0.981	0.986

**Tab. 3.** *In vitro* transcorneal permeation data of moxifloxacin nanosuspension compared with that of marketed formulation (moxicip) through freshly excised goat cornea. Data were expressed as mean ± SD (n=3).

**Formulation**	**Total amount permeated in 4 h (μg ± SD)**	**% Permeation (% ± SD)**	**Corneal Hydration (% ± SD)**
Marketed formulation	134.77 ± 3.23	2.69 ± 0.07	78.30 ± 0.17
MN4	239.29 ± 0.71	4.79 ± 0.01	78.57 ± 0.09
